# *Araucaria heterophylla* oleogum resin essential oil is a novel aldose reductase and butyryl choline esterase enzymes inhibitor: in vitro and in silico evidence

**DOI:** 10.1038/s41598-023-38143-4

**Published:** 2023-07-15

**Authors:** Amal F. Soliman, Mohamed A. Sabry, Gehad Abdelwahab

**Affiliations:** 1 Department of Pharmacognosy, Faculty of Pharmacy, Mansoura University, Mansoura, 35516 Egypt; 2 Department of Medicinal Chemistry, Faculty of Pharmacy, Mansoura University, Mansoura, 35516 Egypt

**Keywords:** Drug discovery, Plant sciences

## Abstract

The essential oil isolated by hydrodistillation of the oleogum resin of *Araucaria heterophylla* has been analyzed by GC–MS. Twenty-four components accounting to 99.89% of the total detected constituents of this essential oil were identified. The major ones were: caryophyllene oxide (14.8%), ( +)-sabinene (12.07%), D-limonene (11.22%), caryophyllene (10.36%), α-copaene (8.00%), *β*-pinene (6.44%), trans-verbenol (5.88%) and *α*-pinene oxide (5.18%). The in vitro inhibitory activities of this oil against aldose reductase, BuCHE, COX-2 and SARS-CoV-2 M^pro^ enzymes were evaluated. This revealed promising inhibitory activity of the essential oil against both aldose reductase and BuCHE enzymes. The molecular docking study of the major components of the *Araucaria heterophylla* essential oil was carried out to correlate their binding modes and affinities for aldose reductase and BuCHE enzymes with the in vitro results. In conclusion, the in vitro inhibitory activity of the essential oil attributed to the synergistic effect between its components and the in silico study suggested that compounds containing epoxide and hydroxyl groups may be responsible for this activity. This study is preliminary screening for the oil to be used as antidiabetic cataract and Alzheimer’s disease therapeutics and further investigations may be required.

## Introduction

The genus *Araucaria* belongs to the family Araucariaceae which is famous for evergreen coniferous ornamental trees. It includes approximately 19 species with limited existence in the southern hemisphere. Several classes of phytoconstituents have been reported in different *Araucaria* species, mainly flavonoids, sesqui- and di-terpenes, and phenylpropanoids compounds. Since ancient times, *Araucaria* species were well known for their medicinal properties as anti-inflammatory, antipyretic, anti-ulcerative, antiviral, antimicrobial, neuro-protective, anti-coagulant, and anti-depressant^1–5^. *Araucaria heterophylla*, one of *Araucaria* species known as ‘Norfolk Island Pine’, was famous for columnar tree used as Christmas tree. Its aerial parts were used in folk medicine for a toothache. It was reported that the chloroform extract of its oleogum resin is rich in labdane diterpenes which exhibited significant antioxidant, antiulcer and anticancer activities. The essential oil (EO) was reported as one of the main constituents of *Araucaria* species. The major compound of the EO derived from the leaves of Australian *A. heterophylla* was α-pinene. Whereas the major compounds of the EO derived from the leaves of Indian *A. heterophylla* were found to be 13-epidolabradiene, beyerene, rimuene, and dolabradiene. While the most abundant compounds identified in the oleogum resin of the same plant were α-copaene, germacrene D, γ-gurjunene, and δ-cadinene^4,6–8^. Up to date, there is only one study of the composition of EO of the oleogum resin of *A. heterophylla* collected from Egyptian culture^4^.

Aldose reductase, a NADPH-dependent oxidoreductase enzyme, is included in reduction of glucose into sorbitol. Large quantities of sorbitol are produced because of the increased flux during hyperglycemia associated with diabetes. As a result of sorbitol deposition and redox imbalance after depletion of NADPH, osmotic stress is produced and causes organ injury and cell damage, which lead to neuropathy, cataract formation and other diabetic complications. Thus, targeting this enzyme is significant in the improvement of diabetes complications^9–11^.

Butyryl cholinesterase (BuChE) and acetyl cholinesterase (AChE) are hydrolysis enzymes for ACh and BuCh neurotransmitters, which have an important role in cognition and memory. These neurotransmitters are observed to be declined in Alzheimer’s disease (AD) patients. Thus, the inhibition of BuChE and AChE enzymes has been a potential treatment for AD^12,13^. Butyrylcholinesterase, which plays a recognized role in the Ach regulation, increases progressively in brain of AD patients while AChE concentration remains unchanged or decreases. Thus, (BuChE)-inhibition can better increase acetylcholine (ACh) levels in treating AD^14,15^.

Cyclooxygenase (COX-2), one of the cyclooxygenase enzymes (COX-1 & COX-2), is an inducible enzyme responsible for producing prostaglandins (PGs) from membrane and releasing arachidonic acid in inflammatory and tumorigenic settings. The dramatic increase of COX-2 expression in inflamed tissue leads to increased prostaglandins synthesis and subsequent development of the cardinal signs of acute inflammation. So, blocking COX-2 enzyme can relieve such signs^16,17^.

SARS-CoV-2 main protease (M^pro^) or (3-chymotrypsin-like protease 3CL^pro^) is an enzyme responsible for the proteolysis and release of essential functioning peptides, playing a great role in replication, and thus the life cycle of the coronaviruses. It is considered one of decisive factors in the infectious route of the virus in addition to papain-like protease (PL^pro^), and RNA-dependent RNA polymerase (RdRp) of SARS-CoV-2 enzymes, which have been reported as important targets for therapeutic strategies^18^.

Nowadays, the use of plant-derived natural products with therapeutic significance attracts great attention due to their availability, affordability and relative safety. This motivates further search into traditional medicine to explore highly effective and safer natural inhibitors for some important enzymes involved in certain disorders and diseases.

In the framework of continuing research on *Araucaria heterophylla* tree, this study is concerned with analyzing the essential oil isolated from the oleogum resin exuded from its trunk. Moreover, discovering potential anti-diabetic cataract, anti-Alzheimer’s disease, anti-inflammatory and anti-COVID-19 leads based on the evaluation of the ability of the major components of this essential oil to inhibit certain enzymes as aldose reductase, BUCHE, COX-2 and SARS-CoV-2 M^pro^ using in vitro and in silico studies.

To the best of our knowledge, this study is the second one to study the composition of EO of the oleogum resin of *A. heterophylla* collected from Egyptian culture. Also, it is the first one to report the inhibitory activity of this essential oil against aldose reductase and BUCHE enzymes.

## Results and discussion

### Identification of the essential oil constituents

The analysis of the essential oil of *Araucaria heterophylla* and the identification of its components were carried out depending on their retention indices^19^, their mass spectral fragmentation patterns^20,21^ and/or stored data on the mass spectral database NIST/ ChemStation data system.

**Figure 1:**
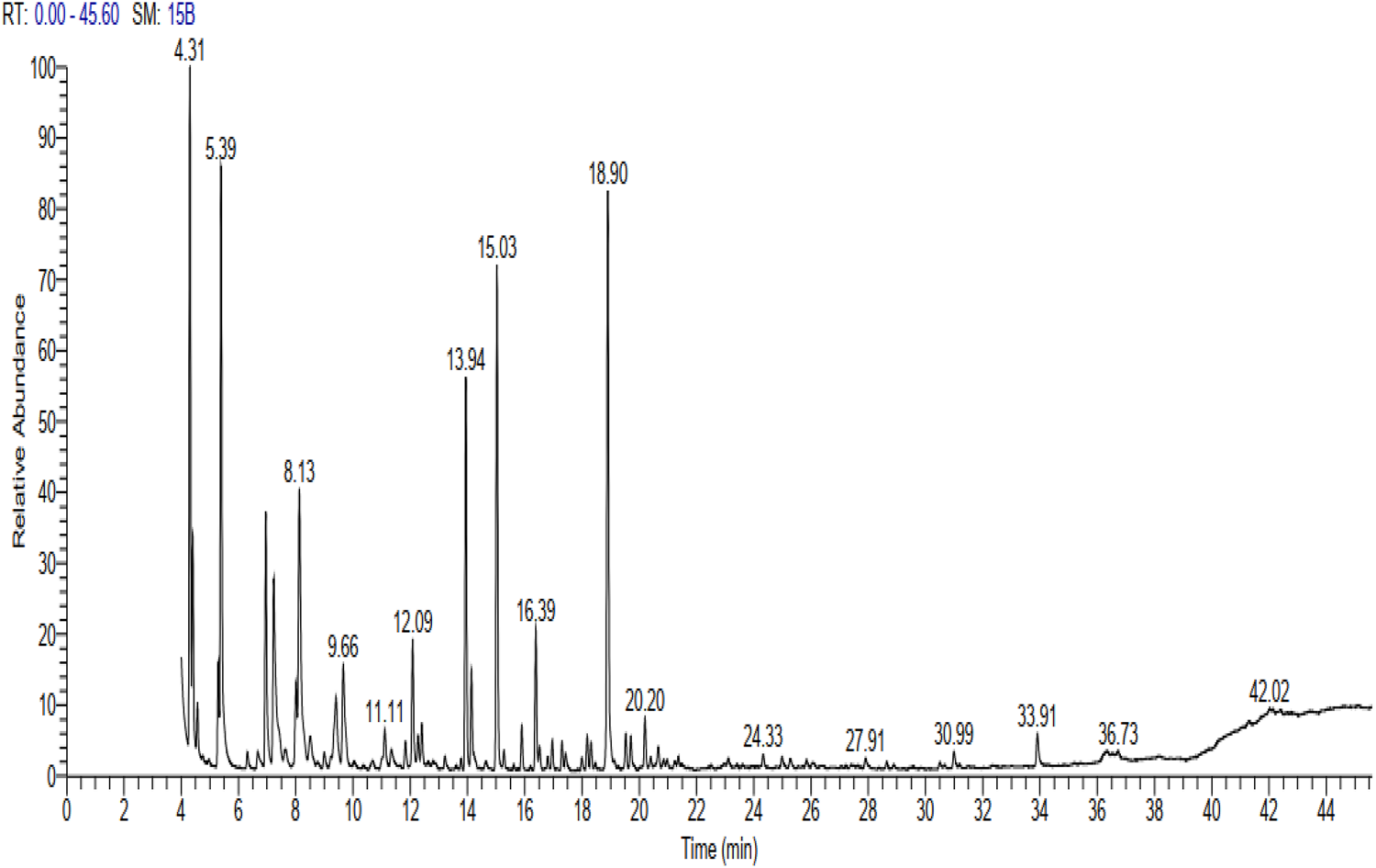
GC–MS chromatogram of essential oil of *Araucaria heterophylla* oleogum resin.

The analysis of the essential oil resulted in the identification of 24 major components representing 99.89% of the total oil composition (Table 1, Fig. 1). The non-oxygenated fraction of this oil was (59.23%). Monoterpenes hydrocarbons (32.93%) were the most abundant non-oxygenated components and ( +)-sabinene (12.07%) was the main constituent followed by D-limonene (11.22%), *β*-pinene (6.44%) in addition to other minors. Caryophyllene (10.36%) was the major sesquiterpene present followed by α-copaene (8.00%). The oxygenated fraction represented about 40.66% in a descending order by oxides (epoxides) and dioxides (22.27%), alcohols (15.50%) and ketones (2.89%). Caryophyllene oxide (14.82%) was the dominant oxide component followed by α-pinene oxide (5.18%). Trans-verbenol (5.88%) was the major alcohol, while levoverbenone was the major ketone present.

**Table 1:** Chemical compositions of the essential oil of *Araucaria heterophylla* oleogum resin.

No	Compound name	Rt	% Area	RI	M^+^ peak	M.F	Base peak
1	( +)- Sabinene	4.30	**12.07**	980	136.2	C_10_H_16_	93.2
2	*β*-pinene	4.40	**6.44**	985	136.2	C_10_H_16_	93.2
3	*β*-Myrcene	4.56	0.88	997	136.3	C_10_H_16_	93.2
4	O-Cymene	5.29	1.70	1011	134.2	C_10_H_14_	119.2
5	D-limonene	5.39	**11.22**	1023	136.2	C_10_H_16_	68
6	α-Pinene oxide	6.95	**5.18**	1143	152.23	C_10_H_16_O	67.1
7	Cis-verbenol	7.23	4.39	1146	152.2	C_10_H_16_O	109.2
8	Trans-pinocarveol	8.00	1.23	1148	152.2	C_10_H_16_O	92.2
9	Trans-Verbenol	8.13	**5.88**	1151	152.2	C_10_H_16_O	109.2
10	(−)-Myrtenol	9.41	2.01	1188	152.23	C_10_H_16_O	79.2
11	Levoverbenone	9.66	2.89	1210	150.2	C_10_H_14_O	107.2
12	Limonene dioxide	11.11	1.52	1144	168.2	C_10_H_16_O_2_	67.1
13	5-Tridecene, (E)	11.83	0.62	1288	182.3	C_13_H_24_	55.2
14	Patchoulane	12.40	0.89	1189	206.36	C_15_H_26_	91.1
15	α-Copaene	13.94	**8.00**	1350	204.35	C_15_H_24_	105.1
16	*β*-bourbonene	14.14	2.06	1380	204.35	C_15_H_24_	81.2
17	Caryophyllene	15.03	**10.36**	1400	204.3	C_15_H_24_	93.1
18	Humulene	15.90	0.91	1442	204.2	C_15_H_24_	93.2
19	γ-Muurolene	16.39	3.49	1480	204.2	C_15_H_24_	161.2
20	γ-Cadinene	17.30	0.59	1510	204.35	C_15_H_24_	161.2
21	Caryophyllene oxide	18.18	**14.82**	1540	220.35	C_15_H_24_O	79.2
22	Germacra-4(15),5,10(14)-triene-1-α-ol	18.31	1.29	1577	220.35	C_15_H_24_O	131.2
23	Humulene oxide II	19.53	0.75	1600	220.35	C_15_H_24_O	67.1
24	( +)-Agathadiol	33.90	0.70	1634	306.48	C_20_H_34_O_2_	81.1
Total %		99.89%					

Non oxygenated compounds	59.23%	Oxygenated compounds	40.66%				
Monoterpenes	32.93%	Monoterpenoids	23.1%				
Sesquiterpenes	26.3%	Sesquiterpenoids	16.86%				
Diterpenes	–	Diterpenoids	0.70%				

Oxygenated compounds	40.66%						
Epoxide and dioxide	22.27%	Alcohols	15.50%	Ketone	2.89%		

Significant values are in bold.

*Rt (min)* retention time, *RI* retention index, *M.F.* molecular formula.

By comparing the composition of EO in our study with previous reported literature^4^, differences in some components and in some percentage of others was found. The EO of the previous study showed that the most abundant component was α-pinene (44.88%) followed by germacrene-D (10.25%), α-copaene (4.72%) and sabinene (4.44%). Also, it showed low percentage of oxygenated components (5.66%) compared to the EO of our study (40.66%). In addition to caryophyllene oxide that exhibited very low percentage (0.33%). In contrast, it was the most abundant component in our study (14.82%).

These variations in the composition of the essential oil could be explained by the impact of extraction technique including solvent, temperature and time of extraction. Also, these variations could be ascribed to the environmental, climatic, genetic factors, age of the plant, soil type or time of harvesting. Thus, it could exert significant effects on the chemical profile of the essential oils derived^4,22,23^.

It was worth mentioning that our plant material was collected from the coastal city, Alexandria while in the previously reported literature, it was collected from Mansoura city in Delta region^4^.

## Enzyme inhibitory activity of the essential oil

### Aldose reductase enzyme inhibition

Up to our knowledge there was no study that evaluated the inhibitory activity of any *Araucaria* species extract or their essential oils against aldose reductase enzyme, thus our study was the first one to report the activity of essential oil of *Araucaria heterophylla* oleogum resin against aldose reductase enzyme. It was found that the essential oil exhibited high significant inhibitory activity against aldose reductase enzyme with potent IC_50_ (0.133 ± 0.006 µg/mL) compared to the control epalrestat (0.165 ± 0.008 µg/mL) (Table 2).

**Table 2:** Aldose reductase, BUCHE, COX-II and SARS-CoV-2 M^pro^ inhibitory activities of the essential oil of *Araucaria heterophylla* oleogum resin compared to standards.

Sample	Aldose reductase IC_50_ µg/mL	Sample	BUCHE IC_50_ µg/mL
EO	0.133 ± 0.006	EO	0.154 ± 0.009
Epalrestat (standard)	0.165 ± 0.008	Rivastigmine (standard)	0.078 ± 0.005

Sample	COX-II IC_50_ µg/mL	Sample	SARS-CoV-2 M^pro^ IC_50_ µg/mL
EO	10.3 ± 0.29	EO	32.1 ± 1.44
Celecoxib (standard)	2.6 ± 0.09	Tipranavir (standard)	4.7 ± 0.21

All data are presented as mean value ± SD for three independent experiments.

### BUCHE enzyme inhibition

It was reported that some essential oils of genus *Araucaria* had inhibitory activities against BUCHE enzyme as the EO of *Araucaria brasiliensis,* so this encouraged us to evaluate the activity of *Araucaria heterophylla* essential oil as inhibitor of BUCHE enzyme^5^. The tested essential oil showed comparable inhibitory activity against BUCHE enzyme with an IC_50_ of 0.154 ± 0.009 µg/mL compared to the control rivastigmine with an IC_50_ of 0.078 ± 0.005 µg/mL (Table 2).

### COX-II enzyme inhibition

In vitro and in silico COX enzyme inhibitory activity was previously reported for *A. heterophylla*, *A. bidwillii* and *A. cunninghamii* leaves extracts, so they could be a possible source of natural anti-inflammatory drugs^24^. The essential oil of *A. heterophylla* resin in our study revealed moderate inhibitory activity against COX-II enzyme with an IC_50_ of 10.3 ± 0.29 µg/mL compared to the control celecoxib with an IC_50_ of 2.6 ± 0.09 µg/mL (Table 2).

### SARS-CoV-2 M^pro^ enzyme inhibition

Previous reported molecular docking study of compounds identified in *A. heterophylla* besides *A. bidwillii* and *A. cunninghamii* leaves extracts revealed possible effect of these compounds against SARS-CoV-2, so they probably have anti-COVID effects^24^. Our essential oil exhibited low inhibitory activity against SARS-CoV-2 M^pro^ enzyme with an IC_50_ of 32.1 ± 1.44 µg/mL compared to the control tipranavir with an IC_50_ of 4.7 ± 0.21 µg/mL (Table 2).

## Molecular docking simulation

As the EO of *A. heterophylla* oleogum resin showed the highest inhibitory activity against aldose reductase and BUCHE enzymes compared to the other two enzymes under investigation, thus molecular docking study was carried out to demonstrate the major 8 components of the EO binding modes and their affinities for these two enzymes, thus exploring the components of the EO responsible for the inhibitory activities against these enzymes and their mode of action.

### Molecular docking simulation against aldose reductase active site

**Figure 2:**
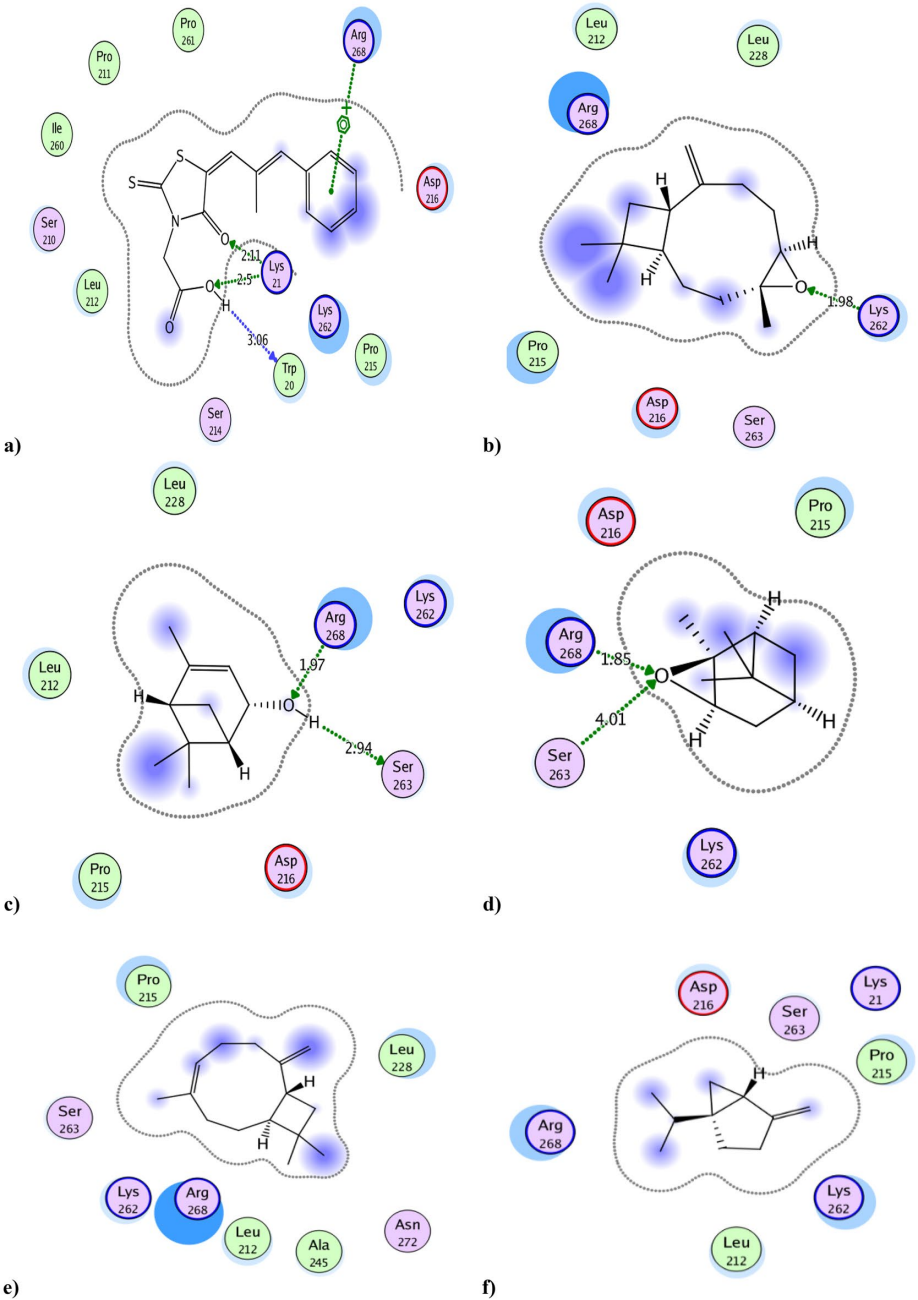

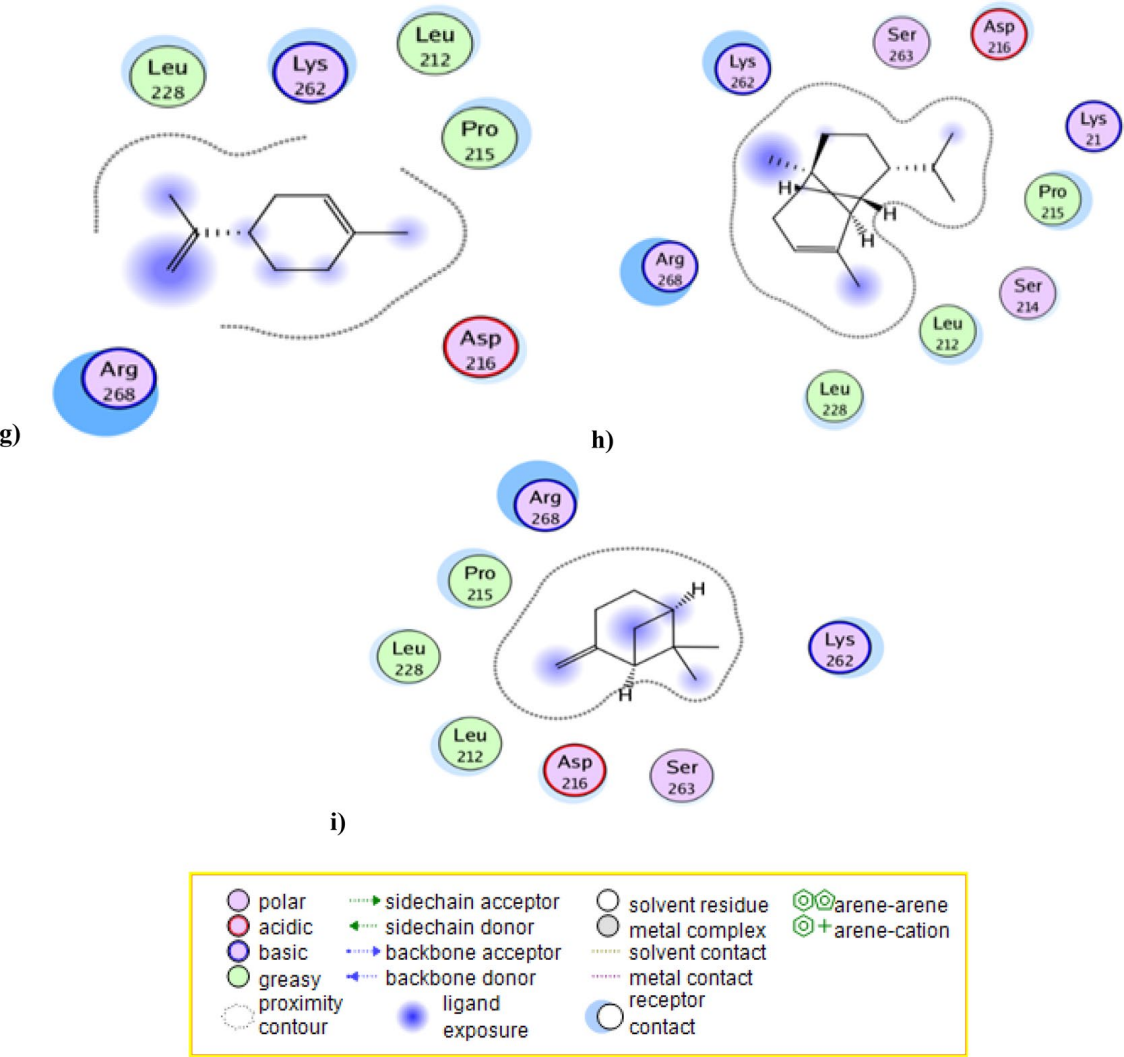
Docking results of the major compounds of the essential oil & the standard epalrestat in the active site of aldose reductase enzyme (3RX2). 2D interactions of (**a**) Standard epalrestat. (**b**) Caryophyllene oxide. (**c**) Trans-verbenol. (**d**) Alpha-pinene oxide. (**e**) Caryophyllene. (**f**) ( +)- Sabinene. (**g**) D-limonene. (**h**) Alpha-Copaene. (**i**) Beta-pinene.

Docking results of the tested essential oil components to aldose reductase active site, using epalrestat as reference inhibitor, showed the binding interaction types and binding scores of essential oil components (Tables 2 and 3 and Fig. 2). The reference epalrestat **(**IC_50_ = 0.165 µg/mL, binding score =  − 11.0 kcal/mol) bound to the active site through its amide and carboxylate moieties by two hydrogen bonds with Lys21 and another H-bond with Trp20, it also bound to active site through arene-cation interaction with Arg268 by its phenyl group. In addition, it formed strong hydrophobic interactions with Lys262, Pro215 and Asp216 amino acid residues.

**Table 3:** The aldose reductase inhibition docking scores^a^ and type of binding interactions of the major components of the essential oil & the standard (Epalrestat).

Comp	Binding energy (Kcal/mol)^a^ (docking score)	Type of binding interactions
Epalrestat	** − 11.0**	Two H-bonds with Lys21
		H-bond with Trp20
		Arene-cation interaction with Arg268 Strong hydrophobic interaction with Lys262, Pro215 and Asp216
Ah major components		
Caryophyllene	− 8.0	Strong hydrophobic interaction with Arg268, Pro215 and Leu228
Caryophyllene oxide	** − 9.0**	H-bond with Lys262
		Strong hydrophobic interaction with Arg268 and Pro215
( +)- Sabinene	− 7.1	Strong hydrophobic interaction with Arg268 and Lys262
D-limonene	− 6.7	Strong hydrophobic interaction with Arg268 and Lys262
Alpha-Copaene	− 8.5	Strong hydrophobic interaction with Arg268 and Lys262
Beta-pinene	− 6.8	Strong hydrophobic interaction with Arg268 and Lys262
Trans-verbenol	** − 7.9**	Two H-bonds with Arg268 and Ser263
		Strong hydrophobic interaction with Pro215 and Lys262
Alpha-pinene oxide	** − 7.7**	Two H-bonds with Arg268 and Ser263
		Strong hydrophobic interaction with Pro215, Asp216 and Lys262

Significant values are in bold.

Epalrestat was used as reference aldose reductase inhibitor compound.

Docking was carried out following the reported procedures^26^ against the aldose reductase enzyme pocket (PDB code ID: 3RX2).

^a^More negative score refers to better capability of a molecule to dock with the target and make more desirable interactions.

Compounds caryophyllene oxide, trans-verbenol and alpha-pinene oxide proved to have the best docking results (binding score values of − 9.0, − 7.9 and − 7.7 kcal/mol, respectively). They shared epalrestat in binding with Arg268, Lys262 and Pro215 by various binding interactions. Caryophyllene oxide bound to active site by its epoxide moiety with Lys262 and other hydrophobic interactions with Arg268 and Pro215. Trans-verbenol and alpha-pinene oxide showed better binding interactions by binding with active site Arg268 and Ser263 amino acid residues with two hydrogen bonds through their hydroxyl and epoxide moieties, respectively. They formed also strong hydrophobic interactions with Pro215 and Lys262 (Tables 2 and 3 and Fig. 2).

Other essential oil major components showed weaker binding interactions with aldose reductase active site. This may be due to their non-polar structure. They only bound to target by hydrophobic bonds. So, it could be predicted that the activity of *Araucaria heterophylla* essential oil against aldose reductase enzyme (IC_50_ = 0.133 µg/mL), that was more potent than the reference epalrestat itself **(**IC_50_ = 0.165 µg/mL) may be attributed to trans-verbenol and alpha-pinene oxide components and in a less extent to caryophyllene oxide.

The other predicted interactions of the tested components with aldose reductase active site were shown in (Table 3, Fig. 2, Tables S1 and S2) and the 3D interactions of the best binding compounds were visualized in (Fig. S1).

### Molecular docking simulation against BuChE active site

**Figure 3:**
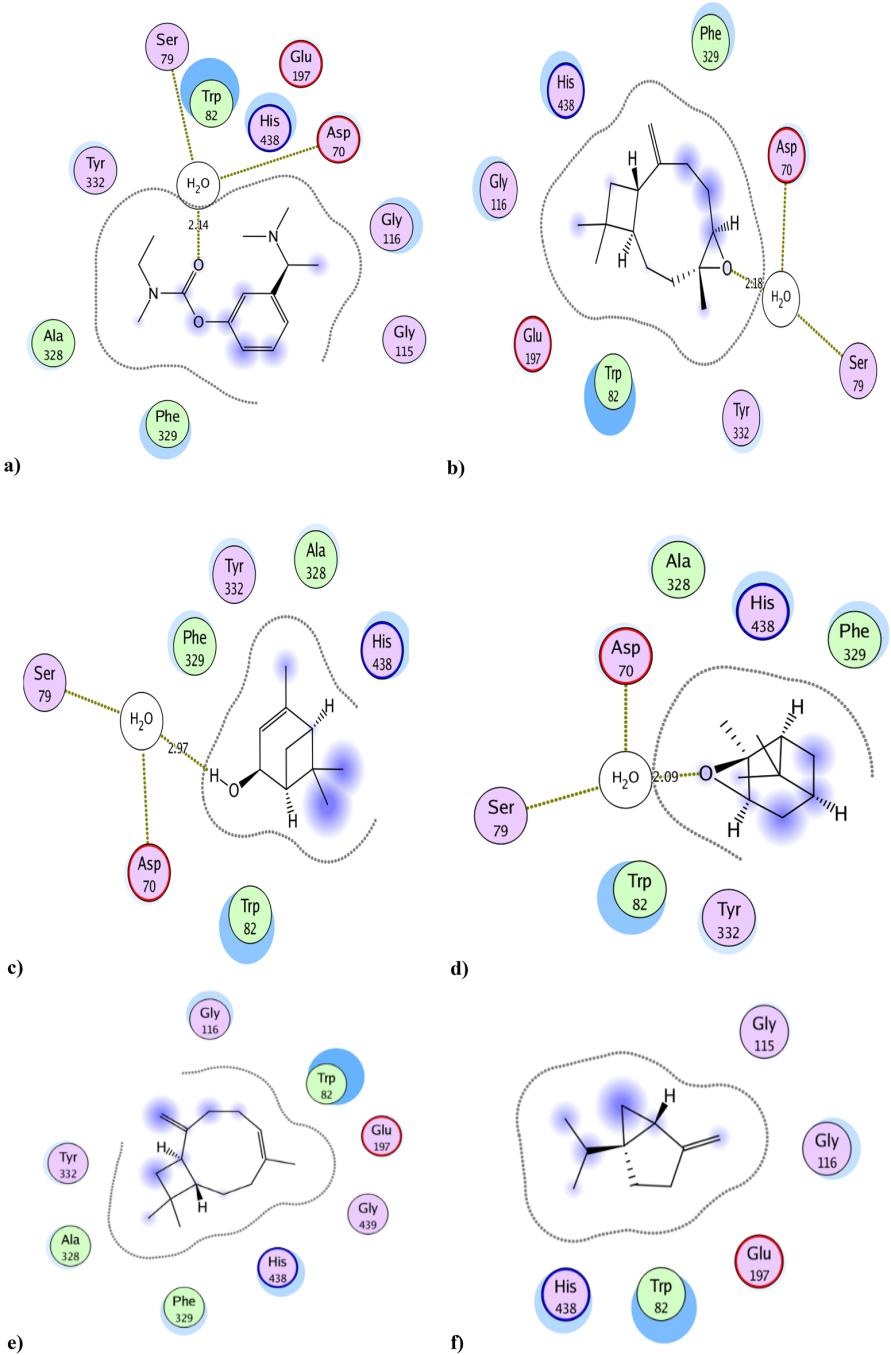

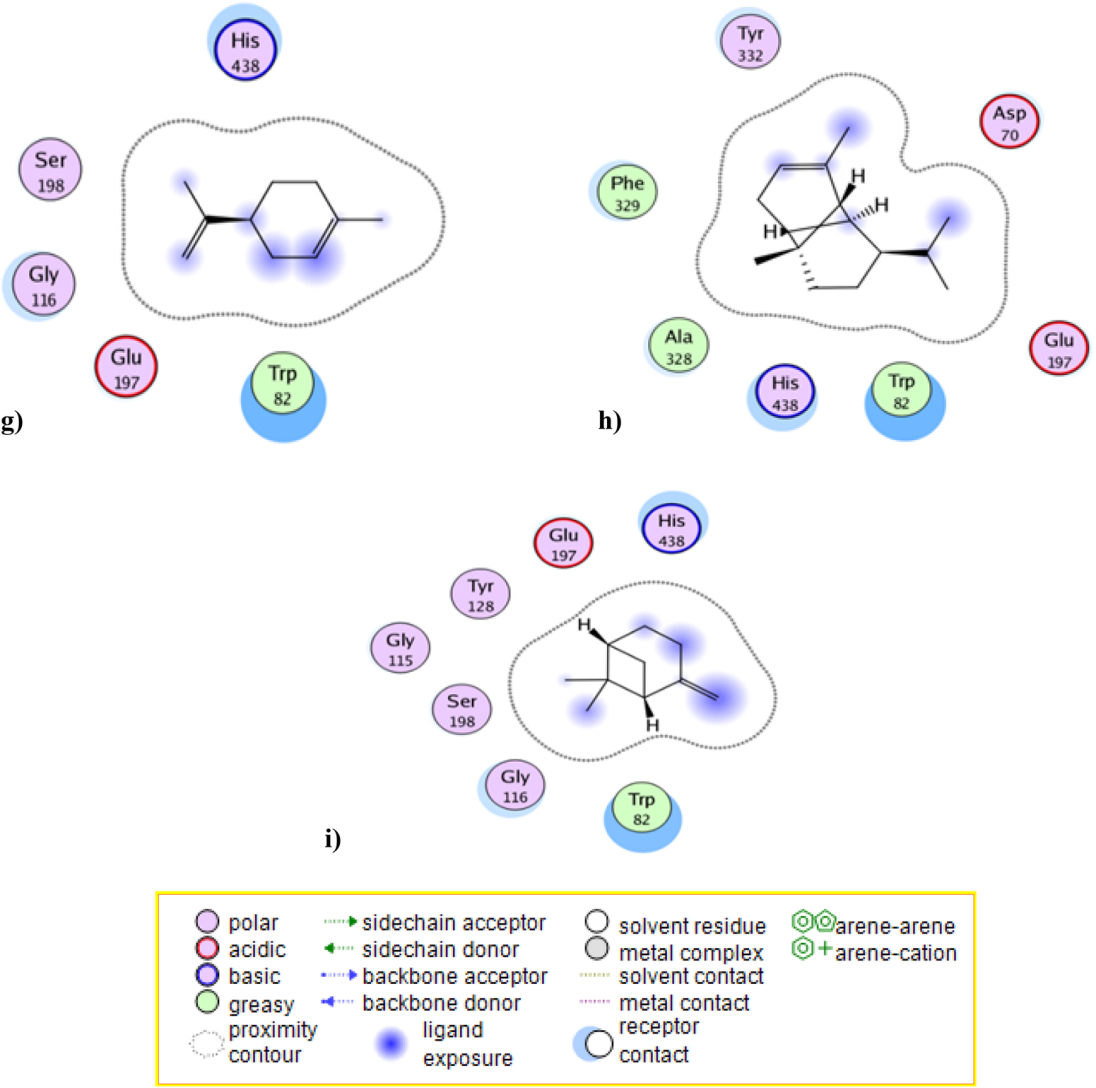
Docking results of the major compounds of the essential oil & the standard rivastigmine in the active site of BuChE enzyme (4BDS). 2D interactions of (**a**) Standard rivastigmine. (**b**) Caryophyllene oxide. (**c**) Trans-verbenol. (**d**) Alpha-pinene oxide. (**e**) Caryophyllene. (**f**) ( +)- Sabinene. (**g**) D-limonene. (**h**) Alpha-Copaene. (**i**) Beta-pinene.

Docking results of the tested compounds to BuChE active site, using rivastigmine as standard inhibitor, showed the types of binding interactions and docking scores of essential oil components. Rivastigmine **(**IC_50_ = 0.078 µg/mL**,** binding score =  − 13.7 kcal/mol) bound to BuChE active site through its carbamate moiety by forming extensive interactions with water molecule strongly anchored to the target by hydrogen bonding to Asp70 and Ser79, the main catalytic amino acids in BuChE enzyme, it bound also to BuChE through strong hydrophobic interaction with Trp82, His438, Gly116 and Phe329 (Tables 2 and 4 and Fig. 3).

**Table 4:** The BuChE inhibition docking scores^a^ and type of binding interactions of the major compounds of the essential oil & the standard (Rivastigmine).

Comp	Binding energy (Kcal/mol)^a^ (docking score)	Type of binding interactions
Rivastigmine	− 13.7	Interaction with a water molecule of the binding site that form hydrogen bond with Asp70 and Ser79
		Strong hydrophobic interaction with Trp82, His438, Gly116 and Phe329
Ah major components		
Caryophyllene	− 8.9	Strong hydrophobic interaction with Trp82 and His438
Caryophyllene oxide	** − 12.0**	Interaction with a water molecule of the binding site that form hydrogen bond with Asp70 and Ser79
		Strong hydrophobic interaction with Trp82, His438 and Phe329
( +)- Sabinene	− 6.6	Strong hydrophobic interaction with Trp82 and His438
D-limonene	− 6.7	Strong hydrophobic interaction with Trp82 and His438
Alpha-Copaene	− 8.6	Strong hydrophobic interaction with Trp82 and His438
Beta-pinene	− 7.0	Strong hydrophobic interaction with Trp82 and His438
Trans-verbenol	** − 7.8**	Interaction with a water molecule of the binding site that form hydrogen bond with Asp70 and Ser79
		Strong hydrophobic interaction with Trp82 and His438
Alpha-pinene oxide	** − 9.5**	Interaction with a water molecule of the binding site that form hydrogen bond with Asp70 and Ser79
		Strong hydrophobic interaction with Trp82, His438 and Phe329

Significant values are in bold.

Rivastigmine was used as reference butyryl choline esterase inhibitor compound.

Docking was carried out following the reported procedures^27^ against the BuChE enzyme pocket (PDB code ID: 4BDS).

^a^More negative score refers to better capability of a molecule to dock with the target and make more desirable interactions.

Compounds caryophyllene oxide, trans-verbenol and alpha-pinene oxide showed the best docking results** (**binding score =  − 12.0, − 7.8 and − 9.5 kcal/mol, respectively). They shared rivastigmine in binding with Asp70 and Ser79 by the same binding manner and also in the strong hydrophobic interaction with almost the same amino acid residues. Caryophyllene oxide and alpha-pinene oxide formed interactions with water molecule anchored to the target by hydrogen bonding to Asp70 and Ser79 through their epoxide moiety, while trans-verbenol formed this interaction through its hydroxyl group (Table 2 and 4 and Fig. 3). To the best of our knowledge, it was reported that caryophyllene oxide exhibited strong cholinesterase inhibitory activities^25^.

Other essential oil major components showed poor interactions with BuChE active site. This may be due to their lipophilic character and absence of any hydrogen bonding functional groups in their structures. They only bound to target by hydrophobic interactions. So, it could be predicted that the BuChE inhibition of essential oil (IC_50_ = 0.154 µg/mL), nearly similar to rivastigmine **(**IC_50_ = 0.078 µg/mL), may be attributed to caryophyllene oxide, trans-verbenol and alpha-pinene oxide components.

The other predicted binding interactions of the tested components with BuChE active site were shown in (Table 4, Fig. 3, Table S3 and S4) and the 3D interactions of the best binding compounds were visualized in (Fig. S2).

From in vitro and in silico study, our findings shed light toward the potential use of the EO from *A. heterophylla* as a green source of aldose reductase and BUCHE inhibitor.

## Conclusion

This study is the first one that reports the in vitro aldose reductase, BUCHE, COX-2 and SARS-CoV-2 M^pro^ inhibitory activities of *Araucaria heterophylla* essential oil. Our findings attracted the attention to the possibilities of using the EO of *A. heterophylla* as a new medicinal natural inhibitor against aldose reductase and BUCHE enzymes as it exhibited promising significant inhibitory activity compared to the standards. Thus, this EO may be suggested for treatment of diabetic cataract as well as Alzheimer’s disease.

## Material and methods

### Plant material

The oleogum resin of cultivated *Araucaria heterophylla* (Salisb.) were collected from the Gardens of Al Montazah, Alexandria, Egypt, in November 2021. The plant identity was confirmed by Associate Prof. Dr Mahmoud Makram Qassem, Department of Vegetables & Floriculture, Faculty of Agriculture, Mansoura University, Egypt. Voucher specimens were coded as Ah-1–2021 and kept in Pharmacognosy Department, Faculty of Pharmacy, Mansoura University, Egypt.

### Essential oil isolation

*Araucaria heterophylla* oleogum resin, weighted 250 g, were subjected after collection, to hydro distillation for 6 h. using a Clevenger-type apparatus based on using water for the extraction of essential oils that were characterized by their hydrophobic nature. The hydrated sample was heated to vaporize volatile constituents, then two layers (aqueous and oil-rich) were produced and oil could be further separated via separating funnels. The hydrodistillation method achieved three main physicochemical processes: hydrodiffusion, hydrolysis and heat decomposition^28^. This extraction was repeated three times to afford 5 ml of essential oil. The oil was dried over anhydrous sodium sulfate then stored at + 4 °C in the dark until tested.

#### For experimental research and field studies on plants

All procedures were conducted in accordance to the relevant institutional, national, and international guidelines and legislation.

### Analysis of essential oil by gas chromatography-mass spectrometry (GC/MS)

GC/MS analysis was carried out using an Agilent 19091S-433 system with a mass selective detector. HP-5 MS capillary column (30 m × 0.25 mm, film thickness: 0.25 μm); injection mode: splitless; split-flow: 10 ml/min; splitless time: 0.80 min; injector and detector temperature: 250 °C; oven temperature was programmed as follows: 60 °C for 2 min and then 5 °C/min programmed at 240 °C; the carrier gas was helium with a constant flow of 1 mL/min; injection volume: 1 µL of diluted essential oil (1% w/v in CH_2_Cl_2_).

### Enzyme inhibitory activity of the essential oil

#### Aldose reductase enzyme inhibition

For measuring the aldose reductase activity: (Catalog # K369-100) colorimetric kit was used. Also, epalrestat was included as a positive control (155 S. Milpitas Blvd., Milpitas, CA 95035 USA, Email: tech@biovision.com).

The aldose reductase inhibitory activity of the essential oil sample was done according to previous literature^10^. The principle of the assay depends on the ability of aldose reductase enzyme to catalyze the oxidation of NADPH to NADP. The absorption of NADPH at 340 nm was measured in a mixture containing NADPH, the enzyme and its substrate in addition to test samples.

#### BUCHE enzyme inhibition

For measuring butyryl choline esterase activity: (Catalog Number EIABCHEF (192 tests) fluorescent kit was used. Also, rivastigmine was included as a positive control (Life Technologies Corporation | Carlsbad, CA 92,008 USA | Toll Free in USA 1 800 955 6288).

The cholinesterase inhibitory activity of the test sample was determined according to the previously published procedure^29^ using Ellman’s microplate spectrophotometric method. The substrate butyryl thiocholine chloride is hydrolyzed by BuChE to give butyrate and thiocholine. In neutral and alkaline media, thiocholine reacts with 5,5-Dithiobis [2-nitrobenzoic acid] (DTNB) to give yellow-colored 2-nitro-5-thiobenzoate, which can be detected spectrophotometrically at 412 nm.

#### COX-II enzyme inhibition

For measuring COX-2 activity: (Catalog # K547-100) fluorometric kit was used. Also, celecoxib was included as a positive control (155 S. Milpitas Blvd., Milpitas, CA 95035 USA, Email: tech@biovision.com).

The Cox-II inhibitory activity of tested sample was carried out according to previous literature^30^. The assay is based on the fluorometric detection of prostaglandin G2, the intermediate product generated from arachidonic acid by the action of COX enzyme at 535/587 nm.

#### SARS-CoV-2 M^pro^ enzyme inhibition

For measuring main protease (SARS-CoV-2): (Catalog #79,955–1) fluorometric kit was used. Also, tipranavir was included as a positive control (6042 Cornerstone Court West, Ste. BSan Diego CA 92,121, Email: info@bpsbioscience.com).

The inhibitory activity against the SARS-CoV-2 main protease (M^pro^ or 3CL^pro^) assay was carried out based on the FRET-based activity assay^31^. The principle of the assay depends on the C-terminal of the peptide substrate being linked to a fluorophor (Edans) and the N-terminal has a fluorescence quencher (Dabcyl) that quenches the fluorescence signal of Edans. Thus, the peptide substrate exhibits low fluorescence because the fluorescence intensity of Edans in the C-terminal is quenched by the Dabcyl in the N-terminal of the substrate.

- Statistical analysis of the data was performed using GraphPad Instat version 8 software package ((GraphPad Software Inc. V8, San Diego, CA, USA). Graphs were sketched using GraphPad Prism. Statistical tests used were one-way analysis of variance (ANOVA) followed by Tukey–Kramer multiple comparisons test for statistical comparison between parametric data.
- The results were expressed as percentage inhibition, which was calculated using the formula:

Inhibitory activity (%) = (1 − A test/A control) × 100.

Where, A test is the reading* in the presence of test substance and A control is the reading* of control. *Absorbance or inflouresence.

### Molecular docking studies

Molecular docking was used as a tool that predicting the binding mode of the essential oil major components with the targeted enzymes. The molecular docking studies in both two- & three-dimensional visualizations were used to predict the binding affinity in Kcal/mol between the essential oil major components and the enzymes active sites, then comparing their results with a reference. Therefore, molecular docking studies helped to pick up the essential oil components that are responsible for the activity. These studies were done only on two enzymes, aldose reductase and BUCHE because of their promising in vitro results.

### Ligand and protein preparation

The essential oil major components structures were adjusted in their least energetic and neutral conformers using the builder of Molecular Operating Environment (MOE) version 2009.10 (Chemical Computing Group Inc. software. https://www.chemcomp.com). They were prepared for docking into the aldose reductase and BuChE active sites. Energy was minimized via MMFF94 technique with root mean square gradient of 0.01 kcal/mol Å. The tested compounds were docked into the active site of crystal structure of human aldose reductase complexed with sulindac sulfone (ID: 3RX2)^26^ and human butyrylcholinesterase in complex with tacrine (BuChE) (ID: 4BDS)^27^. Targets were prepared and active site was detected following the reported procedures^32,33^.

## Supplementary Information

Supplementary Information.

## Data Availability

The datasets used during the current study available from the corresponding author upon request.
