# Supplementary material for: Araucaria heterophylla oleogum resin essential oil is a novel aldose reductase and butyryl choline esterase enzymes inhibitor: in vitro and in silico evidence
Source: Sci Rep. 2023 Jul 15;13:11446. doi: 10.1038/s41598-023-38143-4 (PMC10349848; doi:10.1038/s41598-023-38143-4)
Supplement: Supplementary file 1 — Supplementary Information. [file 41598_2023_38143_MOESM1_ESM.pdf]

Araucaria heterophylla oleogum resin essential oil is a novel aldose reductase and butyryl choline esterase enzymes inhibitor: *in vitro* and *in silico* evidence

Amal F. Soliman[a], Mohamed A. Sabry[b] & Gehad Abdelwahab[a*]

Abstract

The essential oil isolated by hydrodistillation of the oleogum resin of *Araucaria heterophylla* has been analyzed by GC-MS. Twenty-four components accounting to 99.89 % of the total detected constituents of this essential oil were identified. The major ones were: caryophyllene oxide (14.8%), (+)-sabinene (12.07%), D-limonene (11.22%), caryophyllene (10.36%), α-copaene (8.00%), *β*-pinene (6.44%), trans-verbenol (5.88%) and *α*-pinene oxide (5.18%). The *in vitro* inhibitory activities of this oil against aldose reductase, BuCHE, COX-2 and SARS-CoV-2 M^{pro} enzymes were evaluated. This revealed promising inhibitory activity of the essential oil against both aldose reductase and BuCHE enzymes. The molecular docking study of the major components of the *Araucaria heterophylla* essential oil was carried out to correlate their binding modes and affinities for aldose reductase and BuCHE enzymes with the *in vitro* results. In conclusion, the *in vitro* inhibitory activity of the essential oil attributed to the synergistic effect between its components and the *in silico* study suggested that compounds containing epoxide and hydroxyl groups may be responsible for this activity. This study is preliminary screening for the oil to be used as antidiabetic cataract and Alzheimer's disease therapeutics and further investigations may be required.

Keywords: *Araucaria heterophylla*, essential oil, enzymes inhibitor, molecular docking study.

Contents

Tables	Page:
**Table S1:** The aldose reductase inhibition (IC_{50} µg/mL), docking scores[a] and 2D pose of the major components of the essential oil and the reference cor (**Epalrestat**).	**4**
**Table S2:** The aldose reductase inhibition (IC_{50} µg/mL), docking scores[a] and binding interactions of the major components of the essential oil and the reference compound (**Epalrestat**).	**6**
**Table S3.** The BuChE inhibition (IC_{50} µg/mL), docking scores[a] and 2D pose of the major components of the essential oil and the reference compound (**Rivastigmine**).	**8**
**Table S4.** The BuChE inhibition (IC_{50} µg/mL), docking scores[a] and type of interactions of the major components of the essential oil and the reference compou (**Rivastigmine**).	**10**

Figures	Page:
**Fig. S1.** 3D binding mode and residues involved in the recognition of (**a**) **epalrestat**, the compounds showing the best binding interactions (**b**) **caryophyllene oxide, (c) trans-verbenol** and **(d) alpha-pinene oxide** docked and minimized in the aldose reductase binding pocket	**7**
**Fig. S2.** 3D binding mode and residues involved in the recognition of (**a**) **rivastigmine**, the compounds showing the best binding interactions (**b**) **caryophyllene oxide, (c) trans-verbenol** and **(d) alpha-pinene oxide** docked and minimized in the BuChE binding pocket	**11**

Table S1. The aldose reductase inhibition (IC_{50} ug/ml), docking scores[a] and 2D pose of the major components of the essential oil and the reference compound (**Epalrestat**).

Comp.		Aldose reductase inhibition (IC_{50} ug/ml)	Binding energy (Kcal/mol)[a] (docking score)	2D pose
**Ah (Isolated volatile oil)**		0.133 ± 0.006		
**Epalrestat**		0.165 ± 0.008	-11.0	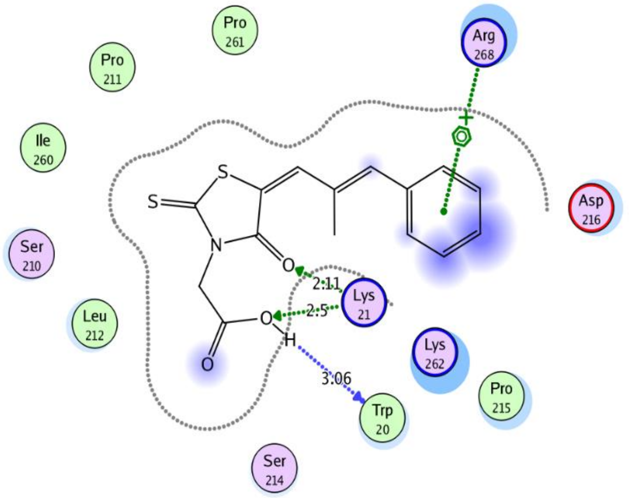
**Ah major components**	**Caryophyllene**	**NT**	-8.0	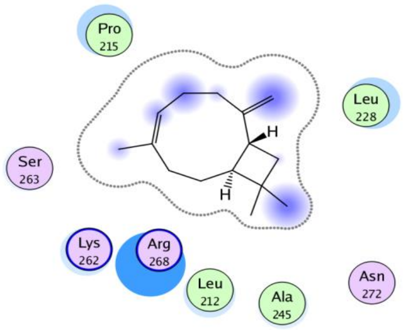
	**Caryophyllene oxide**	**NT**	-9.0	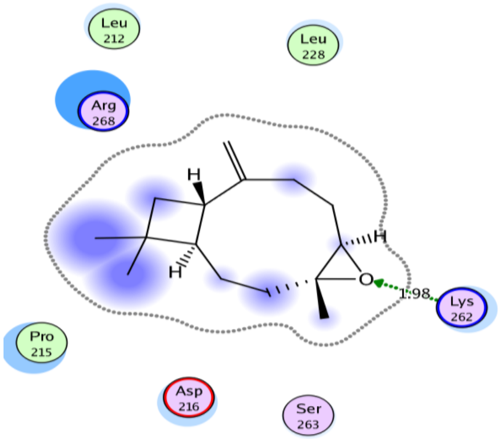

Comp.	Aldose reductase inhibition (IC_{50} ug/ml)	Binding energy (Kcal/mol)[a] (docking score)	2D pose
**(+)- Sabinene**	NT	-7.1	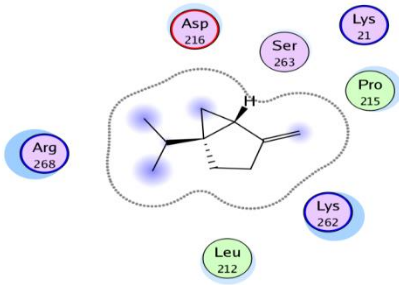
**D-limonene**	NT	-6.7	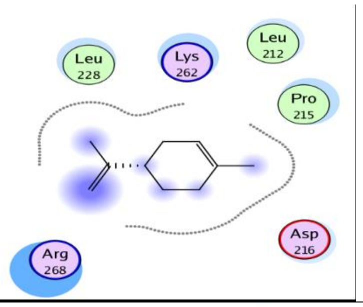
**Alpha-Copaene**	NT	-8.5	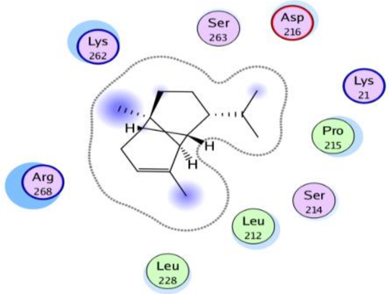
**Beta-pinene**	NT	-6.8	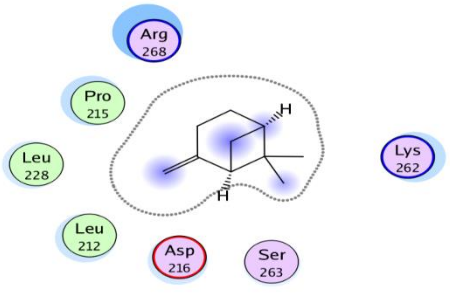
**Trans-verbenol**	NT	-7.9	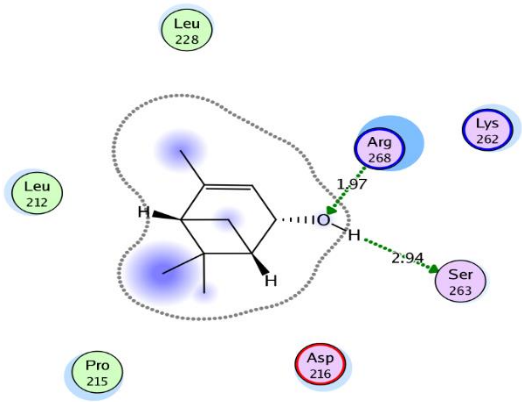

Comp.	Aldose reductase inhibition (IC_{50} ug/ml)	Binding energy (Kcal/mol)[a] (docking score)	2D pose
**Alpha-pinene oxide**	NT	-7.7	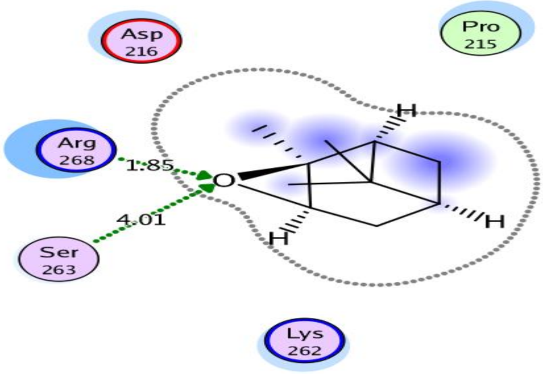

Table S2. The aldose reductase inhibition (IC_{50} ug/ml), docking scores[a] and type of binding interactions of the major components of the essential oil and the reference compound (**Epalrestat**).

	Comp.	aldose reductase **inhibition (IC_{50} ug/ml)**	Binding energy (Kcal/mol)[a] (docking score)	Type of binding interactions
**Ah (Isolated volatile oil)**		0.133 ± 0.006		
**Epalrestat**		0.165 ± 0.008	-11.0	• Two H-bonds with Lys21<br>• H-bond with Trp20<br>• Arene-cation interaction with Arg268<br>Strong hydrophobic interaction with Lys262, Pro215 and Asp216
**Ah major components**	**Caryophyllene**	NT	-8.0	• Strong hydrophobic interaction with Arg268, Pro215 and Leu228
	**Caryophyllene oxide**	NT	-9.0	• H-bond with Lys262<br>• Strong hydrophobic interaction with Arg268 and Pro215
	**(+)- Sabinene**	NT	-7.1	• Strong hydrophobic interaction with Arg268 and Lys262
	**D-limonene**	NT	-6.7	• Strong hydrophobic interaction with Arg268 and Lys262
	**Alpha-Copaene**	NT	-8.5	• Strong hydrophobic interaction with Arg268 and Lys262
	**Beta-pinene**	NT	-6.8	• Strong hydrophobic interaction with Arg268 and Lys262
	**Trans-verbenol**	NT	-7.9	• Two H-bonds with Arg268 and Ser263<br>• Strong hydrophobic interaction with Pro215 and Lys262
	**Alpha-pinene**	NT	-7.7	• Two H-bonds with Arg268 and Ser263<br>• Strong hydrophobic interaction with Pro215, Asp216

Comp.	aldose reductase **inhibition** **(IC_{50} ug/ml)**	**Binding energy (Kcal/mol)[a] (docking score)**	**Type of binding interactions**
**oxide**			and Lys262

- **Epalrestat** was used as reference aldose reductase inhibitor compound.
- All data are presented as mean value ± SD for three independent experiments.
- Abbreviation: **NT**, not tested.
- Docking was carried out following the reported procedure [Zheng, et al., 2012] against the aldose reductase enzyme pocket (PDB code ID: 3RX2)
- [a] More negative score refers to better cabability of a molecule to dock with the target and make more desirable interactions.

Fig. S1. 3D binding mode and residues involved in the recognition of (**a**) **epalrestat**, the compounds showing the best binding interactions (**b**) **caryophyllene oxide, (c) trans-verbenol** and **(d) alpha-pinene oxide** docked and minimized in the aldose reductase binding pocket

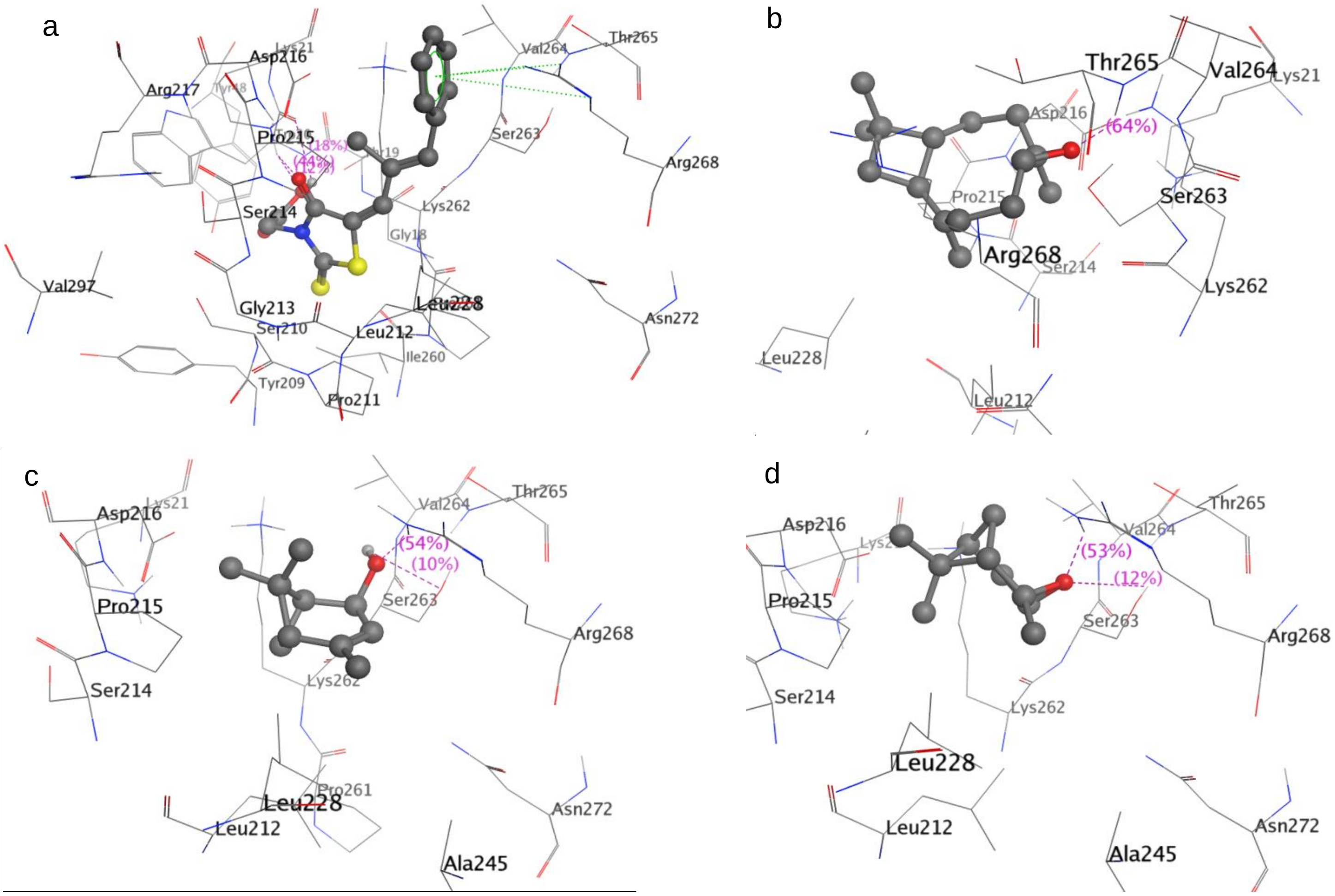

Fig. S1

Table S3. The BuChE inhibition (IC_{50} ug/ml), docking scores[a] and 2D pose of the major components of the essential oil and the reference compound (**Rivastigmine**).

Comp.		BuChE inhibition (IC_{50} ug/ml)	Binding energy (Kcal/mol)[a] (docking score)	2D pose
**Ah (Isolated volatile oil)**		0.154 ± 0.009		
**Rivastigmine**		0.078 ± 0.005	-13.7	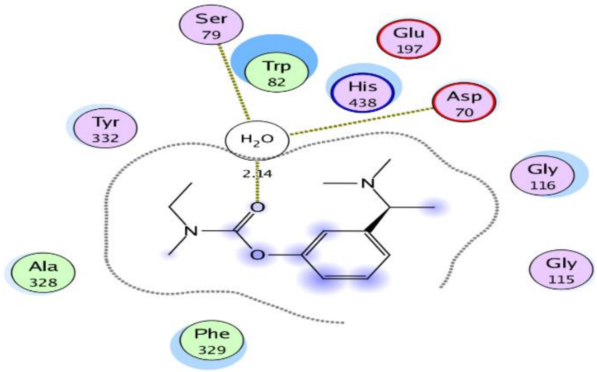
**Ah major components**	**Caryophyllene**	**NT**	-8.9	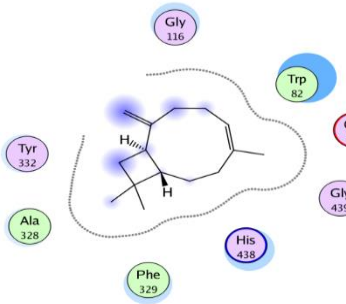
	**Caryophyllene oxide**	**NT**	-12.0	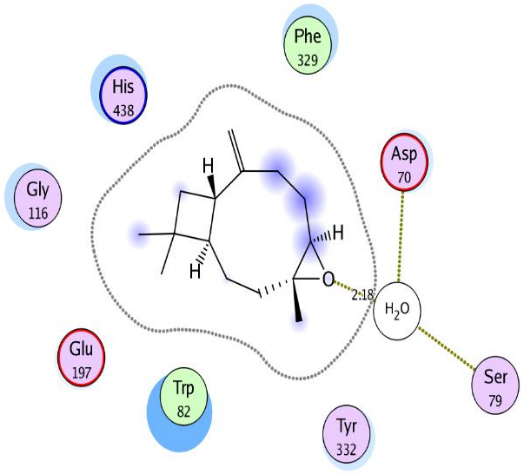
	**(+)- Sabinene**	**NT**	-6.6	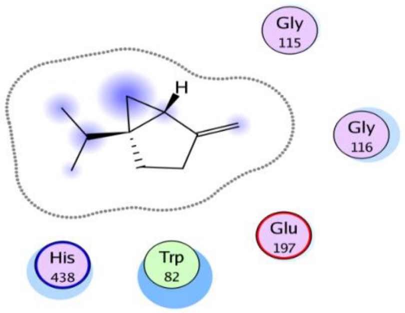

Comp.	BuChE inhibition (IC_{50} ug/ml)	Binding energy (Kcal/mol)[a] (docking score)	2D pose
D-limonene	NT	-6.7	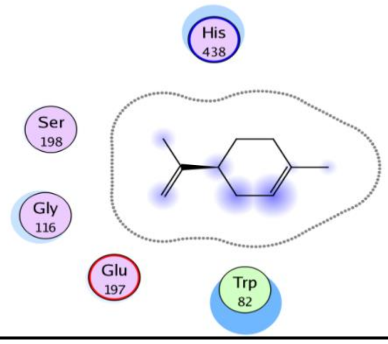
Alpha-Copaene	NT	-8.6	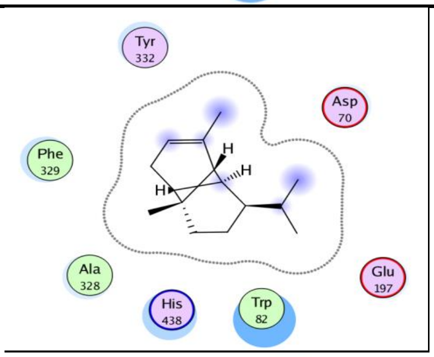
Beta-pinene	NT	-7.0	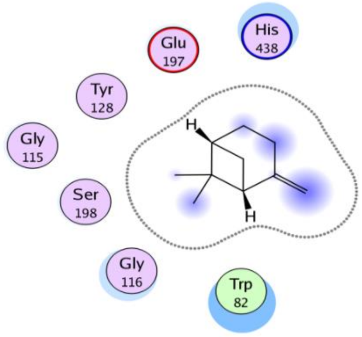
Trans-verbenol	NT	-7.8	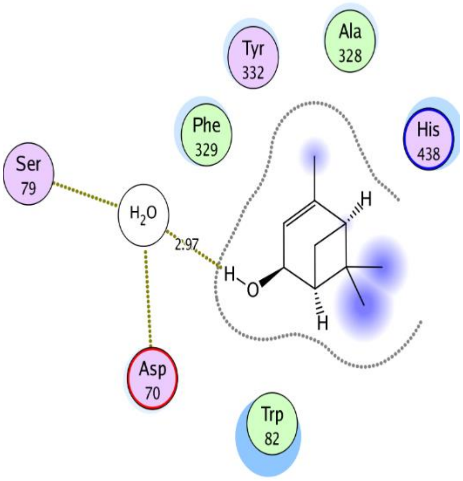

Comp.	BuChE inhibition (IC_{50} ug/ml)	Binding energy (Kcal/mol)[a] (docking score)	2D pose
**Alpha-pinene oxide**	NT	-9.5	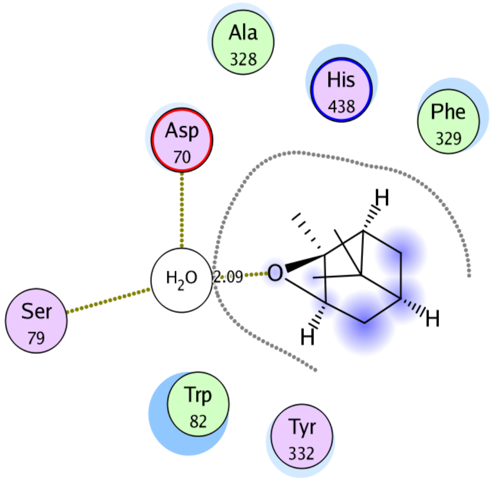

Table S4. The BuChE inhibition (IC_{50} ug/ml), docking scores[a] and type of binding interactions of the major components of the essential oil and the reference compound (**Rivastigmine**).

	Comp.	BuChE inhibition (IC_{50} ug/ml)	Binding energy (Kcal/mol)[a] (docking score)	Type of binding interactions
	**Ah (Isolated volatile oil)**	0.154 ± 0.009		
	**Rivastigmine**	0.078 ± 0.005	-13.7	• Interaction with a water molecule of the binding site that form hydrogen bond with Asp70 and Ser79<br>Strong hydrophobic interaction with Trp82, His438, Gly116 and Phe329
**Ah major components**	**Caryophyllene**	NT	-8.9	• Strong hydrophobic interaction with Trp82 and His438
	**Caryophyllene oxide**	NT	-12.0	• Interaction with a water molecule of the binding site that form hydrogen bond with Asp70 and Ser79<br>• Strong hydrophobic interaction with Trp82, His438 and Phe329
	**(+)- Sabinene**	NT	-6.6	• Strong hydrophobic interaction with Trp82 and His438
	**D-limonene**	NT	-6.7	• Strong hydrophobic interaction with Trp82 and His438
	**Alpha-Copaene**	NT	-8.6	• Strong hydrophobic interaction with Trp82 and His438
	**Beta-pinene**	NT	-7.0	• Strong hydrophobic interaction with Trp82 and His438
	**Trans-verbenol**	NT	-7.8	• Interaction with a water molecule of the binding site that form hydrogen bond with Asp70 and Ser79<br>• Strong hydrophobic interaction with Trp82 and His438

Comp.	BuChE inhibition (IC_{50} ug/ml)	Binding energy (Kcal/mol)[a] (docking score)	Type of binding interactions
**Alpha-pinene oxide**	NT	-9.5	• Interaction with a water molecule of the binding site that form hydrogen bond with Asp70 and Ser79<br>• Strong hydrophobic interaction with Trp82, His438 and Phe329

- **Rivastigmine** was used as reference BuChE inhibitor compound.
- Docking was carried out following the reported procedure [Nachon, et al., 2013] against the BuChE enzyme pocket (PDB code ID: 4BDS)
- All data are presented as mean value ± SD for three independent experiments.
- Abbreviation: **NT**, not tested.
- [a] More negative score refers to better cabability of a molecule to dock with the target and make more desirable interactions.

Fig. S2. 3D binding mode and residues involved in the recognition of (**a**) **rivastigmine**, the compounds showing the best binding interactions (**b**) **caryophyllene oxide, (c) trans-verbenol** and **(d) alpha-pinene oxide** docked and minimized in the BuChE binding pocket

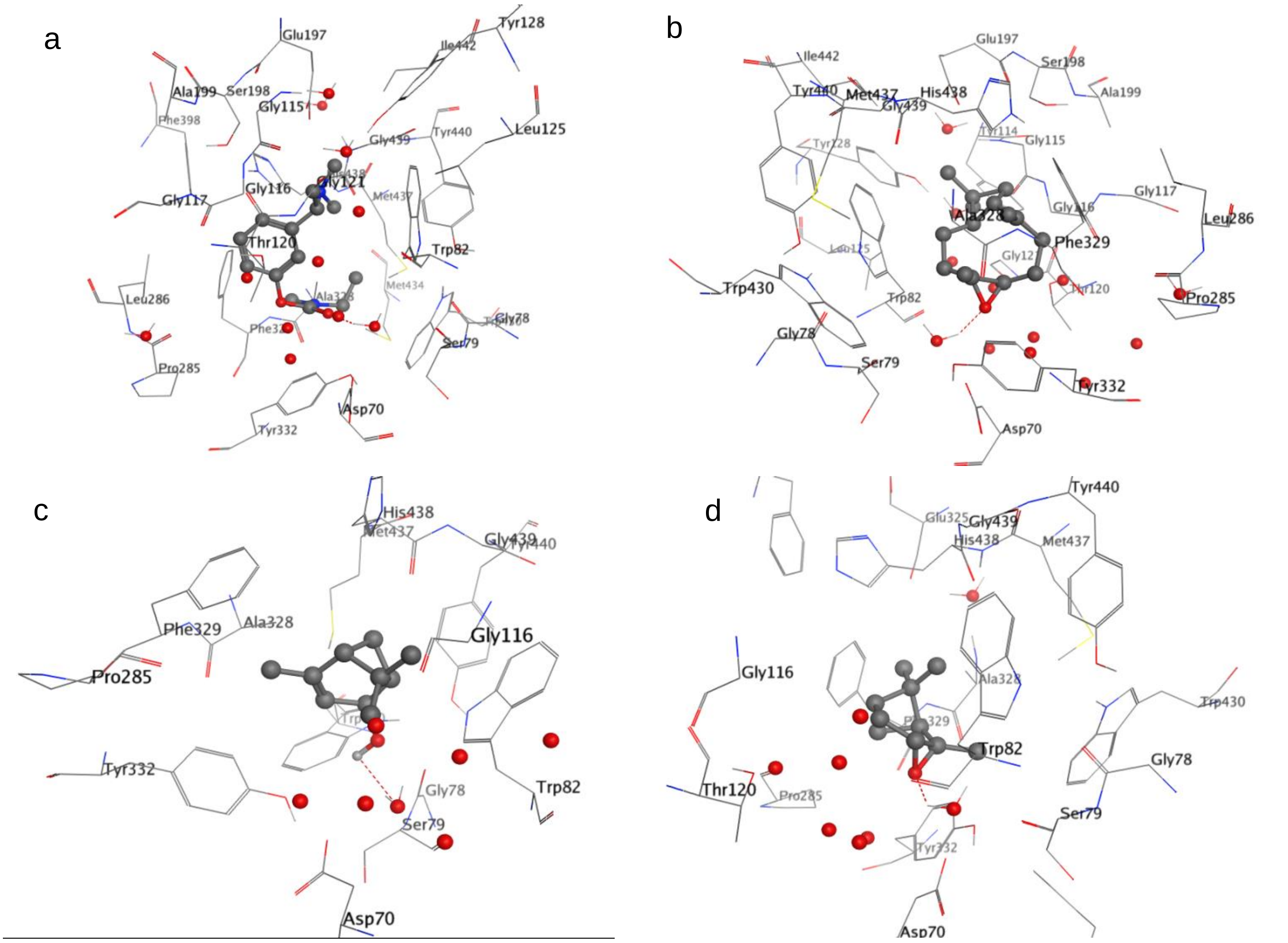

Fig. S2
